# Point-of-care testing in UAE community pharmacies: adoption, operational practices, and quality challenges

**DOI:** 10.3389/fmed.2026.1970393

**Published:** 2026-10-14

**Authors:** Anan S. Jarab, Walid Al-Qerem, Ahmad Z. Al Meslamani, Rahma Elsherif, Hamza Jarab, Omar Jrab, Nada A. Alsaleh, Salahdein Aburuz, Yazid N. Al Hamarneh, Judith Eberhardt

**Affiliations:** 1 Department of Clinical Pharmacy, Faculty of Pharmacy, Jordan University of Science and Technology, Irbid, Jordan; 2 Department of Pharmacy, Faculty of Pharmacy, Al-Zaytoonah University of Jordan, Amman, Jordan; 3 College of Pharmacy, Al Ain University, Abu Dhabi, United Arab Emirates; 4 Faculty of Medicine, Jordan University of Science and Technology, Irbid, Jordan; 5 Barts and the London School of Medicine and Dentistry, Queen Mary University of London, Marsa, Malta; 6 Department of Pharmacy Practice, College of Pharmacy, Princess Nourah Bint Abdulrahman University, Riyadh, Saudi Arabia; 7 Department of Pharmacology and Therapeutics, College of Medicine and Health Sciences, United Arab Emirates University, Al Ain, United Arab Emirates; 8 Department of Pharmacology, Faculty of Medicine and Dentistry, University of Alberta, Edmonton, AB, Canada; 9 Department of Psychology, School of Social Sciences, Humanities and Law, Teesside University, Middlesbrough, United Kingdom

**Keywords:** attitude, barriers, community pharmacist, knowledge, point-of-care testing, practice

## Abstract

**Introduction:**

Community pharmacy-based point-of-care testing can expand access to health screening and chronic disease monitoring, yet evidence on its routine implementation and quality in the United Arab Emirates remains limited. This study assessed service adoption, operational practices, and perceived quality challenges to identify priorities for safe integration into primary care.

**Methods:**

A cross-sectional survey of 501 licensed community pharmacists was conducted using convenience sampling, primarily through in-person visits to community pharmacies, supplemented by electronic dissemination through online platforms and professional networks. The questionnaire examined testing uptake, service types, workflow, data management, training, and implementation challenges. Multivariable logistic regression identified factors associated with providing testing.

**Results:**

Overall, 46.5% of respondents provided at least one point-of-care testing service, and 30.5% of these providers reported always performing testing activities. Male pharmacists had higher odds of providing testing than female pharmacists (adjusted odds ratio 1.49, 95% confidence interval 1.04–2.16). Compared with pharmacists with 1–5 years of experience, those with 6–12 months, 6–10 years, and more than 10 years had lower odds of providing testing. Blood pressure, cholesterol, and glycated hemoglobin testing were the most common services. Only 41.2% of providers integrated results into electronic patient records; 30.9% had received no formal training, and 59.6% of trained providers described their instruction as minimal. The most frequently reported quality problems were insufficient patient information (90.6%), improper sample collection (90.1%), and equipment malfunction (89.7%).

**Discussion:**

By linking service uptake with workflow and quality risks, this study provides baseline evidence that point-of-care testing is established in many community pharmacies but lacks consistent supporting infrastructure. Standardized competency programs, operating procedures, and interoperable records are needed to improve reliability, support safe expansion, and strengthen the contribution of community pharmacies to accessible primary care**.**

## 1 Introduction

Point-of-care testing (POCT) can be defined as diagnostic tests conducted at or near the site of patient care, yielding immediate results that enable prompt clinical decisions (1). By providing on-the-spot health screenings and disease monitoring, POCT enables pharmacists in community settings to take on a more direct role in patient care, going beyond simply dispensing products. POCT in pharmacies has grown in popularity worldwide within the last five years. For example, in the United States the number of community pharmacies that can perform onsite lab tests increased by 173% between 2015 and 2023 (2).

Beyond its increasing availability, the potential value of community pharmacy-based POCT lies in its ability to support timely clinical decision-making and improve patient outcomes when integrated with pharmacist-led clinical management. A systematic review found that it reduced inappropriate antimalarial treatment, although its effects on glycated hemoglobin, lipid, and international normalized ratio control were mixed or inconclusive (3). More recently, trained Canadian pharmacists using point-of-care international normalized ratio testing and decision-support software achieved high-quality warfarin control, with a mean time in therapeutic range of 74.2% (4).

Customers and shop providers welcomed the use of POCT in private retail settings, despite some health workers' reservations (5, 6). In Canada, the New Brunswick College of Pharmacists used a systematic literature review, nationwide regulatory scan, and stakeholder survey to draft a “right-touch” policy that (i) initially limits pharmacy-led POCT to chronic-disease medication management; (ii) mandates written standard operating procedures (SOPs), device calibration, and external quality-assurance logs; and (iii) formally integrates trained pharmacy technicians (7).

Despite evidence supporting its feasibility in community pharmacy practice, POCT implementation continues to face barriers at multiple levels. At the pharmacy level, insufficient training, time, staffing, suitable space, and integration with existing workflows and electronic health records can limit service delivery. At the health-system and stakeholder levels, inadequate remuneration, unclear clinical responsibility, limited physician collaboration, patient concerns, and regulatory requirements may hinder adoption (8, 9). For respiratory infection testing specifically, patient needs and resources, external policies and incentives, and the perceived relative advantage of POCT were prominent implementation considerations (8). Consistent with these findings, a US study during COVID-19 found that 59% of pharmacists identified workflow impact as a barrier, followed by lack of payment mechanisms (55%) and insufficient training (21%) (10).

POCT implementation challenges have been reported internationally, but their nature and importance may vary according to healthcare systems, regulatory contexts, and pharmacy practice environments. In the United Arab Emirates (UAE), the integration of POCT in community pharmacies is still emerging, and data on current practices are limited. The current studies suggest that community pharmacists in the UAE are receptive to expanding their services (11–14). A study showed that 71.8% of pharmacists “strongly agreed” that pharmacies in the UAE should provide extended services, and many were willing to offer basic POCT-related services such as blood pressure checks and pregnancy testing (15). On the regulatory front, health authorities have begun to lay the groundwork for pharmacy-based POCT. For example, under defined scope-of-practice standards, the Department of Health–Abu Dhabi now permits community pharmacists to perform certain non-diagnostic tests and screenings: finger-prick blood glucose, glycated hemoglobin (HbA1c), cholesterol, international normalized ratio (INR) testing (16).

Our previous study examined community pharmacists' knowledge, attitudes, and concerns regarding POCT in the UAE (17). The present study builds on this work by shifting the focus from perceptions to practice, specifically exploring the adoption of POCT services, operational procedures, and quality-related challenges within community pharmacy settings. By addressing these implementation and quality dimensions, this study offers new insights into the practical integration of POCT in UAE community pharmacies that were not covered previously. This study aims to assess the prevalence of POCT uptake in community pharmacies in the UAE and to describe UAE community pharmacies' operational methods and quality issues related to these services. The findings will provide policymakers and stakeholders with baseline data, guiding future integration of POCT into community pharmacy practice in the UAE.

## 2 Materials and methods

### 2.1 Study site and subjects

This study is part of a larger project that initially aimed to assess pharmacists' knowledge, attitudes, and practices regarding POCT. The current phase focuses on evaluating the prevalence, operational practices, and perceived quality challenges of POCT in community pharmacies across the UAE. Eligible participants were licensed pharmacists currently practicing in community pharmacies who had graduated from universities recognized by the UAE Ministry of Higher Education and were registered with the UAE Ministry of Health. Pharmacists not currently practicing in community pharmacies, pharmacy students, interns, and individuals who did not provide informed consent were excluded. Participants were recruited using convenience sampling between September 2024 and January 2025, primarily through in-person visits to community pharmacies. During these visits, a research pharmacist explained the study, assessed eligibility, and provided qualifying pharmacists with a link to a self-administered questionnaire hosted on Google Forms. In order to achieve broad geographic reach across the UAE, the link was also circulated electronically through online platforms and professional networks to broaden recruitment. Before beginning the questionnaire, participants were presented with an introductory statement outlining the purpose of the study and emphasizing that participation was voluntary, anonymity would be protected, and all responses would remain confidential. Participants provided electronic informed consent by selecting the statement, “I have read the study information and I agree to participate,” before proceeding to the questionnaire. Completing the questionnaire took approximately 10 min.

### 2.2 Ethical approval

Ethical approval for this research was granted from the research ethics committee at Al Ain University- Abu-Dhabi Campus (Ref #: COP/AREC/AD/05) in August^5^th, 2024.

### 2.3 Study tool

A structured, self-administered questionnaire was developed from relevant research studies (18–20). The instrument consisted of four major sections designed to comprehensively capture pharmacist experiences and insights. The first section collected sociodemographic and professional characteristics such as age, gender, level of education, years of pharmacy experience, and the average number of prescriptions dispensed daily, which helped contextualize the respondents' backgrounds. The second section assessed POCT adoption and usage patterns, including whether pharmacists currently practice POCT, the frequency of use, and their primary motivations for offering such services, such as enhancing pharmacy services, meeting patient demand, improving patient care, or increasing revenue. This section also included a list of POCT services commonly offered, allowing participants to select one or more types of tests they perform, which is presented in Figure 1: Types and frequency of POCT tests reported by pharmacists**.** The third section focused on day-to-day operational aspects of POCT. It addressed who performed the tests (pharmacists, technicians, or external providers), how data from POCT was recorded and managed (e.g., integrated electronic systems, manual logging, or not saved), the frequency of equipment maintenance and calibration, and the extent of staff training. Respondents were also asked about the staff-to-device ratio and the nature of training provided categorized as minimal, basic/single session, or comprehensive/certified. The fourth section explored challenges encountered during POCT implementation. Participants identified specific issues such as errors in sample collection, reagent expiry, equipment malfunction, time constraints, and discrepancies with laboratory results. These challenges were visually summarized and analyzed in Figure 2, which consists of several components: Figure 2A identifies the leading causes of errors related to improper sample collection; Figure 2B highlights the causes of deficiencies in quality control measures; and Figure 2C outlines contributing factors to errors caused by insufficient or inaccurate patient information. Each of these figures was based on multiple-response questions, where pharmacists could select more than one contributing factor, thereby capturing the complexity of operational difficulties in POCT services.

**Figure 1:**
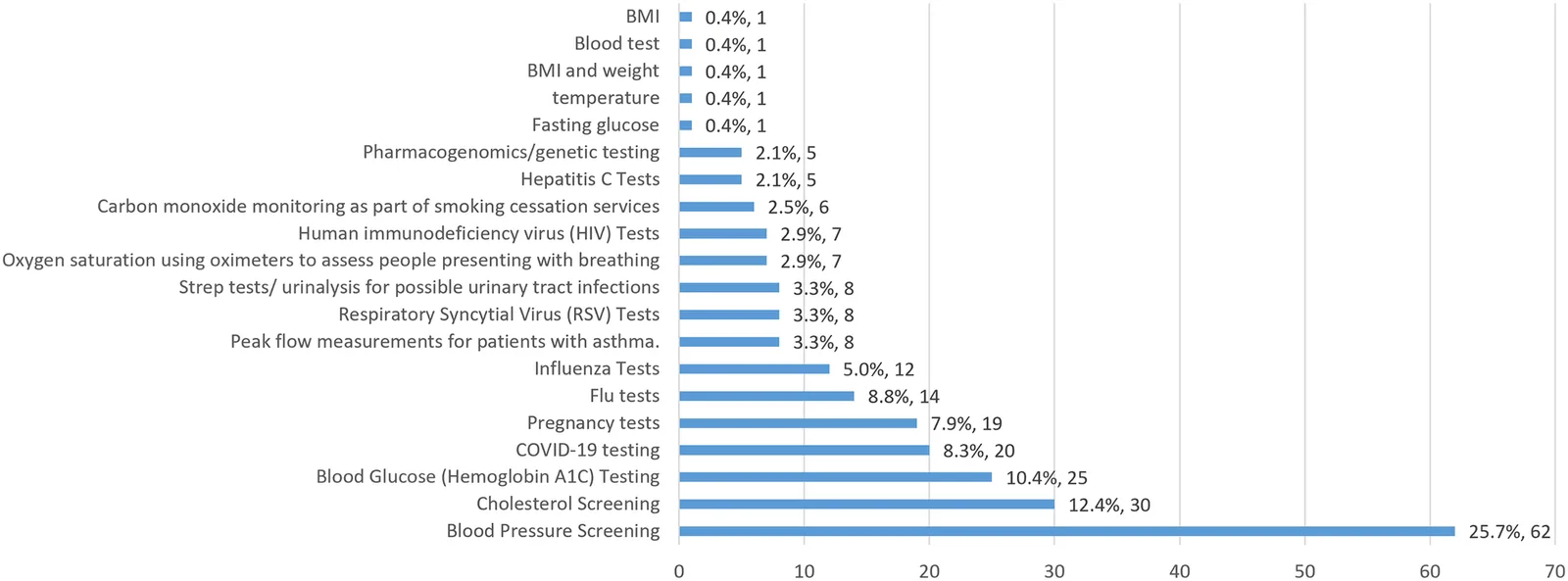
Types and frequency of POCT tests reported by pharmacists. Participants could select more than one response.

**Figure 2:**
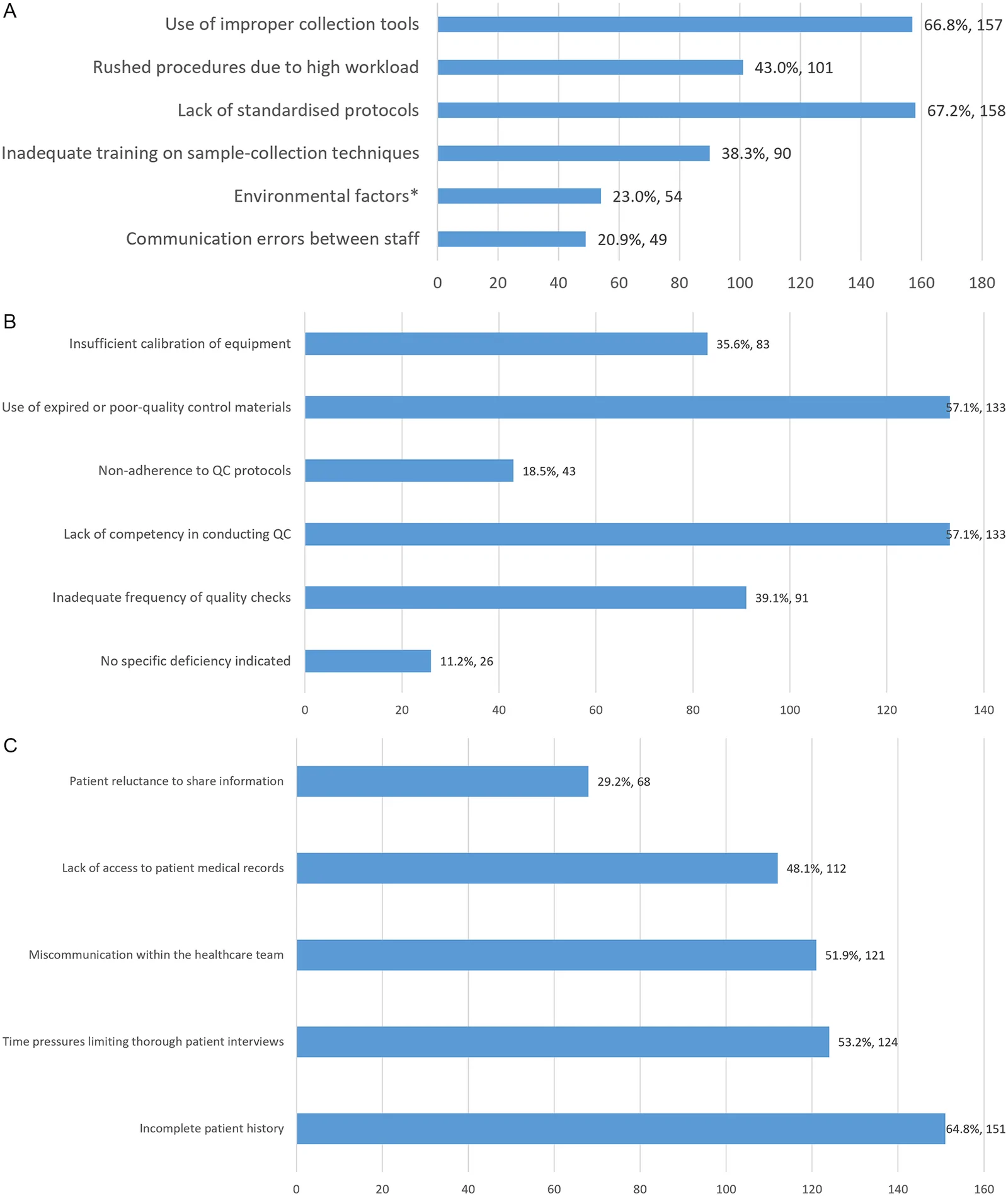
Problems encountered during point-of-care testing operations among pharmacists: **(A)** causes of sample-collection errors; **(B)** causes of deficiencies in quality-control measures; and **(C)** factors contributing to errors associated with insufficient patient information. Participants could select more than one response. *Environmental factors include temperature and humidity.

### 2.4 Instrument validity and reliability

Content validity was assessed through qualitative expert review by a panel of five professionals with expertise in pharmacy practice, clinical diagnostics, and health services research. The panel reviewed each individual questionnaire item for relevance, clarity, comprehensiveness, and alignment with the study objectives, and their feedback informed minor revisions to wording, content, and flow of the questionnaire. Because this validation process involved qualitative appraisal rather than numerical item-level ratings, an item-level content validity index (I-CVI) and formal panel agreement statistic were not calculated. The revised questionnaire was subsequently pilot-tested with 20 community pharmacists to assess its clarity, comprehensibility, and practical applicability. The pilot findings were not included in the final sample. The reliability of the knowledge and attitude scales was confirmed by Cronbach's alpha values of 0.76 and 0.81, respectively.

### 2.5 Sample size calculations

To determine the appropriate sample size for this study, we applied the standard formula for sample size calculation as outlined by Pourhoseingholi (21), Vahedi, and Rahimzadeh (2013): *n* = (Z^2^ × p × (1—p))/d^2^. In this formula, *p* denotes the estimated proportion of the population, which we set at 0.5 to reflect the highest possible variability. The margin of error (*d*) was set at 0.05, and we used a *Z*-value of 1.96, corresponding to a 95% confidence level. Based on these parameters, the minimum required sample size was calculated to be 385 participants. To account for potential non-responses and incomplete questionnaires, we recruited a total of 501 pharmacists.

### 2.6 Statistical analysis

Data were analysed using IBM SPSS Statistics for Windows, version 26.0 (IBM Corp., Armonk, NY, USA). Bivariate associations between practising POCT (yes/no) and each categorical predictor (e.g., sex, experience strata, qualification, daily prescription load) were examined with Pearson's *χ*^2^ or Fisher's exact test when an expected cell count was <5. A *P*-value threshold of 0.20 was used during bivariate screening to reduce the likelihood of excluding potentially important predictors, consistent with methodological recommendations (22). Variables meeting this criterion were entered into a multivariable binary logistic regression model to estimate adjusted odds ratios (AORs) and 95% confidence intervals for independent predictors of POCT practice. The final model was built using forward-stepwise likelihood-ratio selection. Model fit was assessed using the Hosmer–Lemeshow goodness-of-fit test (*P* = 0.136), which indicated no evidence of poor fit. The final model was built with forward-stepwise likelihood-ratio selection. Goodness-of-fit verified with the Hosmer–Lemeshow test (*p* = 0.136), which indicates adequate fit.

## 3 Results

A total of 501 eligible pharmacists provided consent and completed the survey. Of the respondents, 233 (46.5%) were older than 35 years and 276 (55.1%) were male (Table 1). 35 The number of pharmacists who reported practicing POCT was 233 (46.5%), of which 71 (30.5%) reported that they “always” perform POCT operations.

**Table 1:** Sociodemographic information of the study sample (*n* = 501).

Item	Total, *n* (%)
Age (years)	
22–26	122 (24.3%)
27–35	147 (29.3%)
>35	232 (46.3%)
Sex	
Male	276 (55.1%)
Female	225 (44.9%)
Education	
BSc in pharmacy	400 (79.8%)
Doctor of Pharmacy	60 (12.0%)
Master's degree or PhD	41 (8.2%)
Years of experience	
6 months to 1 year	55 (11.0%)
1–5 years	169 (33.7%)
More than 5 years	277 (55.3%)
Prescriptions dispensed per day	
Fewer than 10	141 (28.1%)
More than 10	360 (71.9%)
Practicing POCT	
Yes	233 (46.5%)
No	268 (53.5%)
Frequency of practicing POCT^a^	
Always	71 (30.5%)
Usually	94 (40.3%)
Sometimes	53 (22.7%)
Rarely	15 (6.4%)

a Denominator is 233.

As shown in Table 2, male pharmacists were more likely than females to conduct POCT (AOR: 1.49; 95% CI: 1.04–2.16; *p* = 0.029). Relative to those with 1–5-year of experience, the adjusted odds ratios of performing POCT were lower for pharmacists with 6 months up to 1 year of experience (AOR: 0.36; 95% CI: 0.20–0.64; *P* = 0.001), 6–10 years (AOR: 0.31; 95% CI: 0.14–0.69; *P* = 0.004), and >10 years (AOR: 0.47; 95% CI: 0.27–0.81; *P* = 0.007).

**Table 2:** Factors associated with POCT practicing.

Independent variable	Categories	AOR (95% CI)	*P* value
Age (years)	Continuous	0.987 (0.964–1.1011	0.2217
Gender	Female (Ref)	1.00	
	Male	1.489 (1.043–2.157)	0.029
Years of experience	1–5 years	1.000	
	6 months	0.357 (0.199–0.640)	0.001
	6–10 years	0.311 (0.140–0.691)	0.004
	More than 10 years	0.465 (0.267–0.809)	0.007

AOR, adjusted odds ratio.

The top three POCT tests reported by pharmacists were blood pressure screening (62, 25.7%), Cholesterol screening (30, 12.4%), and HBA1c testing (25, 10.4%) (Figure 1). Pharmacists reported adopting POCT primarily to enhance pharmacy services 167 (71.7%) and meet patient demand 140 (60.1%) (Table 3). Among pharmacists who practice POCT, 96 (41.2%) integrate POCT results into electronic patient profiles, 68 (29.2%) still rely on paper logs, 44 (18.9%) record only informal notes, and 25 (10.7%) do not store results at all. More than half (129, 55.4%) of those practicing POCT are performing the tests themselves. Around one-third of the pharmacists (72, 30.9%) who practice POCT reported that they did not receive formal training on POCT. Among those who were trained (*n* = 161), 96 (59.6%) rated depth of instructions on POCT as minimal.

**Table 3:** A summary of POCT utilization and training among pharmacists (*n* = 233).

Item	Total, *n* (%)
Reasons for practicing POCT	
Enhance pharmacy services	167 (71.7%)
Meet patient demand	140 (60.1%)
Improve patient care	111 (47.6%)
Increase pharmacy revenue	79 (33.9%)
Management of POCT data	
Electronic records integrated with patient profiles	96 (41.2%)
Manual logging	68 (29.2%)
Informal notes	44 (18.9%)
Data are not saved	25 (10.7%)
POCT tests are performed by	
The pharmacist	129 (55.4%)
A technician	92 (39.5%)
Another facility	12 (5.2%)
Frequency of maintenance and calibration for POCT equipment	
Daily	62 (26.6%)
Weekly	91 (39.1%)
Monthly	68 (29.2%)
Rarely	11 (4.7%)
Never	1 (0.4%)
Received formal training on POCT	
Yes	161 (69.1%)
No	72 (30.9%)
Ratio of trained staff to POCT devices available in the pharmacy	
1/1	96 (41.2%)
2/1	58 (24.9%)
≥3	27 (11.6%)
No specific ratio	52 (22.3%)
Level of POCT training provided to the pharmacy staff^a^	
Minimal	96 (59.6%)
Basic/Single-Session	41 (25.5%)
Comprehensive/Certified	24 (14.9%)

a Denominator is 161.

The top three problems reported by pharmacists who practice POCT were errors linked to insufficient patient information 211 (90.6%), improper sample collection 210 (90.1%), and equipment malfunction 209 (89.7%) (Table 4). By contrast, the least frequently reported issues involved difficulties training new staff owing to the diversity of POCT devices 175 (75.1%) and errors arising from insufficient POCT training 143 (61.4%).

**Table 4:** Problems encountered during POCT operations (*n* = 233).

Problem encountered	Total, *n* (%)
Inaccurate POCT results due to inadequate quality control measures	
Yes	199 (85.4%)
No	34 (14.6%)
Incorrect results due to improper sample collection in POCT	
Yes	210 (90.1%)
No	23 (9.9%)
Equipment malfunction while conducting POCT	
Yes	209 (89.7%)
No	24 (10.3%)
Issues in POCT accuracy due to the use of expired reagents or kits	
Yes	183 (78.5%)
No	50 (21.5%)
Errors in POCT due to insufficient patient information	
Yes	211 (90.6%)
No	22 (9.4%)
Errors in POCT due to time constraints	
Yes	192 (82.4%)
No	41 (17.6%)
Discrepancies between POCT results and laboratory findings	
Yes	187 (80.3%)
No	46 (19.7%)
Delays in POCT results due to equipment shortages	
Yes	193 (82.8%)
No	40 (17.2%)
Variability in POCT outcomes due to environmental conditions (e.g, temperature, humidity)	
Yes	201 (86.3%)
No	32 (13.7%)
Difficulties in training new staff on POCT due to the diversity of equipment used	
Yes	175 (75.1%)
No	58 (24.9%)
Errors in POCT due to lack of training on POCT	
Yes	143 (61.4%)
No	90 (38.6%)

In term of sample collection errors, the most commonly reported reasons included the use of improper tools 157 (66.8%) and lack of standardised protocols 158 (67.2%) (Figure 2A). In terms of equipment malfunction and quality measures deficiencies, use of expired or poor-quality materials 133 (57.1%) and non-adherence to protocols 133 (57.1%) were the most frequently reported driving factors (Figure 2B). Incomplete patient history 151 (64.8%) was the most frequently reported factor contributing to error in POCT due to problems in patient information (Figure 2C).

## 4 Discussion

Pharmacy practice has been evolving and POCT becomes a necessary development to improve patient care and accessibility (11, 23, 24). This study quantifies the prevalence of POCT practicing among UAE community pharmacists and describes technical, training, and quality measures including problems encountered during operating these services.

This study found that 46.5% of community pharmacists reported practicing POCT, with about 30.5% of these always conducting such tests. While many countries have approved the adoption of POCT in community pharmacies and provided specific guidelines, real world data about the exact uptake of POCT are limited. In the US, a 2020 survey in Wisconsin showed only 17% of community pharmacies offered POCT at that time (25). The number of pharmacies with licenses to perform simple lab tests nearly doubled from 2019 to 2021, and by 2023 had reached almost 30,000 sites (roughly 50% of pharmacies) (26). In the Middle East, pharmacists' involvement in health screening has historically been limited, a 2011 Qatari study found over 70% of the public did not expect community pharmacists to perform any health screening (27).

This study reported significant demographic disparities in POCT engagement. Male pharmacists were more likely than female pharmacists to practice POCT. This finding points to a gender gap in service adoption, though the reasons are likely multifactorial. It may reflect differences in workplace roles or opportunities—for example, male pharmacists might more often hold positions, such as pharmacy managers or owners of independent pharmacies) that facilitate implementing new services, whereas female pharmacists could be disproportionately represented in settings or job roles with fewer POCT opportunities. It is important to note that literature does not suggest any lesser willingness or capability among female pharmacists *per se*. On the contrary, a Middle Eastern study on pharmacists' professional roles reported “no gaps” in male vs. female pharmacists' enthusiasm to extend their roles in patient care (28). Additionally, our results demonstrated that pharmacists with 1–5 years of experience were most likely to undertake POCT, whereas those with less experience (new practitioners with 6–12 months) and those with greater experience (more than 5 years, including 6–10 and >10 years) were considerably less likely to participate. This pattern suggests an “early-career sweet spot” for implementing cutting-edge services. Because clinical skills and point-of-care diagnostics have become increasingly emphasized in pharmacy curricula and continuing education, pharmacists in their first few years post-qualification may have received recent training in these areas. After about a year of practice, they likely acquire the confidence and practical workflow integration required to provide POCT routinely. Conversely, those new to the profession may still be acclimating to core responsibilities and therefore may be less confident or have fewer opportunities to perform POCT. More experienced pharmacists (with 10 years or more in practice) may be less inclined, or have less time, to implement new services, as they are often more entrenched in traditional dispensing or management duties. This aligns with research indicating that heavy workloads and ingrained routines in busy pharmacies can inhibit the adoption of novel practices (29). Thus, our study aligns with broader findings that younger, highly qualified pharmacists are leading emerging clinical services, whereas both very junior and veteran practitioners participate less. These trends highlight the need for targeted strategies, such as workflow modifications, mentoring programs, and incentives, to help pharmacists from diverse demographic backgrounds embrace POCT and to ensure that training and motivation are applied consistently.

The findings of the current study show that cardiometabolic screening tests, including blood pressure, cholesterol, and HbA1c are the most frequently deployed pharmacy-led POCT. This could be explained by the high prevalence of hypertension, dyslipidaemia, and diabetes in the UAE (30–32). The relatively low cost of these tests could be also a contributing factor to this finding. A nationwide in-pharmacy screening in 297 Norwegian community pharmacies revealed that 19% of adults were unaware they had elevated total cholesterol, highlighting the public-health value of pharmacy access to such tests (33). Additionally, this study found that 41% of UAE pharmacists interface results with electronic profiles while nearly 40% still rely on paper or informal notes underscores a well-recognised connectivity gap; international guidance stresses that poor device-to-EHR integration hampers data reconciliation and quality oversight in multi-site programmes (34). Almost one-third of POCT providers in our study lacked formal instruction and 60% of the remainder judged their training “minimal”, which is consistent with several studies and multinational audits that identify inadequate clinical training as a principal barrier to scaling pharmacy POCT services (10). To address this, Pope et al., suggested a standardized technician-training program, which increased POCT screenings by 46% (35). This underscores how robust, systematic training is critical for scaling pharmacy-based POCT services.

The most errors reported by pharmacists in this study were insufficient patient information, improper sample collection, and equipment malfunction. These same problem areas are emphasised in CLIA-based quality-control guidance for POCT, where sample-tracking, verified controls, and operator competency are mandated to minimise inaccuracies, yet prior work has discussed them mainly in regulatory or commentary form rather than through pharmacy-specific data (35). Simundic examined pre-analytical POCT mistakes and emphasised under-filled tubes, capillary “milking,” and patient-identification errors as frequent risks when non-laboratory workers conduct tests under time constraints (36). She also called for process standardisation and ongoing staff training. By empirically demonstrating the prevalence of these risks in community pharmacies, our study highlights the need for robust QC procedures, thorough training, and practical competency checks in the pharmacy setting. This shifts the conversation from theoretical requirements to gaps in real-world practice.‏

The pharmacists included in this study identified several common causes, including the use of improper tools, a lack of standardised sample-collection protocols, expired or poor-quality reagents, non-adherence to written procedures and incomplete patient histories.‏ Strong SOPs, real-time inventory control to prevent the use of expired materials, and automated result capture are essential for reliable POCT performance, according to international quality frameworks (ISO 15189/22870) and AACC connectivity guidance (37). Failure in any of these areas increases the risk of transcription errors and analytical drift.‏ A review article found that use of suboptimal or expired materials are primary sources of POCT errors (38). However, due to lack of empirical data, the review relied on guidelines and regulatory reports, for example, a POCT guideline from the Canadian Society of Clinical Chemists highlights clinicians' non-compliance with established policies and procedures as the primary threat to test quality and lists quality indicators that can be used to track this gap (39).

Importantly, these findings indicate that the operational weaknesses are interconnected: incomplete patient information may affect test selection and interpretation, inadequate documentation may disrupt follow-up, and limited training may compound sample-collection and equipment-related errors. Addressing any one component in isolation may therefore be insufficient. Drawing on the framework proposed by Alshorman and Hussain, the UAE could adopt a coordinated governance model linking scope-of-practice requirements, competency assessment, standardized documentation, quality monitoring, confirmatory-testing criteria, and referral pathways (40). This approach would help transform POCT from an isolated transaction into a traceable clinical service integrated with primary care.

### 4.1 Study limitations

Recall bias and social-desirability effects may affect this cross-sectional study, which relies on pharmacists' self-reported practices to capture POCT activity at a single point in time. Because the survey link was also circulated through open online platforms and professional networks, the number of pharmacists who received the invitation could not be determined; consequently, a conventional response rate could not be calculated, limiting assessment of potential nonresponse bias. Although recruitment was intended to achieve broad geographic reach across the UAE, emirate of practice was not collected as a participant characteristic; therefore, participation could not be tabulated by emirate and emirate-specific variation in POCT practice could not be assessed.

## 5 Conclusion

POCT use is widespread but inconsistent in UAE community pharmacies, with early-career male pharmacists providing a limited range of cardiometabolic tests. Significant weaknesses in quality management are revealed by training deficiencies, disjointed paperwork, and recurring pre-analytical mistakes. Addressing these issues through organised competency programmes, standard operating procedures, and interoperable data platforms could increase test accuracy, integrate POCT into pharmacy workflows, and improve patient outcomes. These baseline findings can inform policymakers as they support workforce development, refine regulations, and embed pharmacy-based POCT within national chronic-disease initiatives.‏

## Data Availability

The original contributions presented in the study are included in the article/Supplementary Material, further inquiries can be directed to the corresponding author/s.
